# “Everyone else gets ice cream here more often than I do—It burns me up” - Perspectives on Diabetes Care from Nursing Home Residents and their Doctors

**DOI:** 10.1186/s12877-016-0199-0

**Published:** 2016-01-26

**Authors:** Caroline Barnhart, Keelan McClymont, Alex K. Smith, Alvin Au-Yeung, Sei J. Lee

**Affiliations:** University of California, San Francisco, CA USA; San Francisco VA Medical Center, Division of Geriatrics, University of California San Francisco, 4150 Clement St, Bldg 1, Room 220 F, San Francisco, CA 94121 USA

**Keywords:** Diabetes mellitus, Quality of Life, Geriatrics, Fingerstick

## Abstract

**Background:**

To explore the perspectives of nursing home (NH) residents with diabetes and their doctors regarding the burdens of living with diabetes and diabetes treatments.

**Methods:**

Qualitative study of nursing home residents aged 65 and older with diabetes (*n* = 14) and nursing home physicians (*n* = 9) at a Department of Veterans Affairs nursing home (known as the Community Living Center). A semi-structured interview was used to elicit nursing home residents’ and physicians’ perspectives on the burden of diabetes and diabetes treatments. Transcripts were analyzed using constant comparative methods.

**Results:**

The mean age of the nursing home residents was 74; Most (93 %) were male and 50 % self-identified themselves as white. The mean age of nursing home physicians was 39 and 55 % were geriatricians. Dietary restrictions, loss of independence and fingersticks/insulin were noted to be the most burdensome aspects of diabetes. Nursing home residents with a more positive outlook were generally more engaged in their care, while nursing home residents with a more pessimistic outlook were less engaged, allowing their physicians to assume complete control of their care. While physicians noted the potential negative impact of dietary restrictions, nursing home residents’ comments suggest that physicians underestimate the burden of dietary restrictions.

**Conclusions:**

Veterans Affairs nursing home residents were substantially burdened by their diabetes treatments, especially dietary restrictions and fingerstick monitoring. Since there is little evidence that dietary restrictions improve outcomes, fewer dietary restrictions may be appropriate and lead to lower treatment burdens for nursing home residents with diabetes.

**Electronic supplementary material:**

The online version of this article (doi:10.1186/s12877-016-0199-0) contains supplementary material, which is available to authorized users.

## Background

A large and growing proportion of older adults residing in nursing homes (NHs) in the United States have diabetes mellitus. In 2009, 8.4 million older Americans had diabetes; in 2034, 14.6 million older Americans are projected to have diabetes [1]. Among older adults residing in NHs in 2007, nearly one third had diabetes [2]. The prevalence of diabetes among veterans was nearly double the prevalence in non-veterans in 2000, suggesting that diabetes is especially common among elderly Veterans Affairs (VA) NH residents [3, 4].

Despite the high prevalence of diabetes among older adults, there have been relatively few studies focusing on this rapidly growing population and even fewer studies focusing on elders residing in US NHs [5–7]. As a result, little is known about older NH residents’ perceptions of the burdens of having diabetes and their perceptions of the burdens of diabetes treatments. A recent study suggests that the net benefit (or harm) of glycemic treatment is very sensitive to a patient’s views of treatment burden [8]. This suggests that our current knowledge gap of NH residents’ views of disease and treatment burden may result in suboptimal treatment decisions.

Therefore, we conducted a qualitative study to explore NH residents’ and NH physicians’ perspectives on 1) living with diabetes and 2) burdens of diabetes treatments. We focused on older veterans with diabetes mellitus (DM) living in the San Francisco Veterans Affairs Nursing Home (known as the Community Living Center or CLC). Since physicians play a large role in treatment decisions, we also sought to ascertain whether physicians’ perspectives differed from NH residents’ perspectives.

## Methods

### Study design and sample

Between July 2012 and July 2014, we conducted semi-structured, in-depth qualitative interviews with older veterans residing in the San Francisco Veterans Affairs Nursing Home. We also interviewed VA NH physicians to gain their perspective on the experiences of NH residents with diabetes. NH residents aged 65 and older with a diagnosis of Diabetes Mellitus who passed the Mini-Cog screening test (three item recall and clock draw) were eligible to participate. Diagnosis of diabetes mellitus was determined by an HbA1c result of >6.5 % within the past year, or receipt of glucose-lowering medications, such as insulin, metformin, glipizide or glyburide. Eligible patients were identified through chart review and those who passed the Mini-Cog screening test were invited to share their perspectives.

### Data collection

After obtaining informed consent, 14 VA NH residents and 9 VA NH physicians were interviewed (~45 minutes) using English language interview guides. Both interview guides were designed to elicit NH residents’ and NH physicians’ perspectives on the experience of NH residents with diabetes. Specifically, VA NH residents were asked open-ended questions about their experiences living with diabetes, followed-up with additional questions asking residents to describe their feelings about the diabetes care they receive, what influenced their treatment decisions, and what their treatment goals were (See Additional file 1 for complete interview guide). Physicians were asked open-ended questions about how they made glycemic treatment decisions for their patients, their perceptions of the burdens their patients faced, and how they helped their patients reach their treatment goals (See Additional file 2 for complete interview guide).

### Data analysis

All interviews were audio-recorded, transcribed and then analyzed using NVivo 10. Data were analyzed using constant comparative methods and were reviewed iteratively to identify new themes [9–11]. Two researchers (KM and CEB) individually coded a subset of transcripts (*n* = 3) and then held team meetings to compare and develop a coding framework. The researchers then coded the remainder of the transcripts using the coding framework, resolving all coding disagreements through discussion. Codes were added as new themes emerged throughout the coding process and prior transcripts were recoded to reflect the new themes. We stopped enrolling additional NH residents and physicians when no new themes were emerging, suggesting that thematic saturation had been reached.

After this process of open coding, we conducted axial coding, linking categories that had emerged [12]. Finally, we conducted selective coding, to form the storyline of the perceptions of diabetes and diabetes treatments in the nursing home [12]. The Committee on Human Research of the University of California, San Francisco and the San Francisco Veterans Affairs Research and Development Committee approved this study. Participants provided written informed consent.

## Results

### Characteristics of participants

The mean age of the VA NH residents was 74; 13 of 14 were male (93 %). Seven residents self-identified themselves as white (50 %), three self-identified as black (21 %), one self-identified as Asian (7 %), and three were unknown or declined to answer (21 %). 11 (79 %) NH residents required insulin. (Table 1)

**Table 1:** Characteristics of CLC Residents (N = 14) and Physician Respondents (N = 9)

Characteristics	N (%)
*Resident Characteristics*	
Age, mean (range)	74 (66–86)
Female Gender	1 (7 %)
Race/Ethnicity	
African American	3 (21 %)
Caucasian	7 (50 %)
Asian	1 (7 %)
Unknown/refused to answer	3 (21 %)
Requiring insulin	11 (79 %)
*Physician Characteristics*	
Age, mean (range)	39 (31–50)
Female Gender	6 (67 %)
Race/Ethnicity	
African American	0 (0 %)
Caucasian	6 (67 %)
Asian	2 (22 %)
Unknown/refused to answer	1 (11 %)
Specialty	
Family Practice	1 (11 %)
Internal Medicine	3 (33 %)
Geriatrics	5 (55 %)
Years of Practice, mean (range)	11 (4–23)

The mean age of the VA NH physicians was 39 and 6 (67 %) were female. Five physicians were trained in geriatric medicine and four were trained in internal medicine or family practice. This cohort of physicians had been practicing for a mean of 11 years (range: 4–23 yrs). (Table 1)

### Theme 1: Overall burdens of having diabetes

All VA NH residents and physicians talked about the burden of having diabetes and how diabetes lowered quality of life. (Table 2) One older veteran (#10, male, 66) went so far as to state that having diabetes was *“kind of like being in prison.”* Most physicians recognized that a substantial proportion of the burden of diabetes stemmed from treatments. One geriatrician (#4, Female, 32) noted, *“Sometimes when we’re prescribing all that we don’t appreciate how big a burden it is.”*

**Table 2:** Burdens of Living with Diabetes in the Nursing Home

Themes	Representative CLC Resident Quotes	Representative Physician Quotes
General comments about diabetes and diabetes treatments	“In general, [diabetes has] been the pits. It’s got me slowed down.” (#15, Male, 73) “It’s just not being able to do everything that I used to do and…not being able to [eat] all the stuff I used to eat.” (#4, Male, 68)	“I think we underplay the burdens and overestimate the benefits [of treatment.]” (#6, Male, 47)
Dietary restrictions and the diabetic diet	“I have a nasty sweet tooth; as soon as I figure out which one it is I’d probably pull it.” (#2, Male, 66) ”You just can’t go out after a movie and have a milkshake and it means being constantly aware that there [is this thing] to be dealt with.” (#13, Male, 69) “Well I don’t like the food they put in here period; it’s putrid and it’s–I don’t eat what they give me, I eat other food” (#5, Male, 79) “I can’t have sweets and fattening food and stuff like that you know? That was all the food I like.” (#4, Male, 68). “All I have to do is stay away from anything with sugar in it and I don’t do that real well either. I figure they’re just going to stick me with more insulin anyway. But I know I can have sugar; I just can’t sit there and eat a pound of it at a time. So I get little bits of it—just enough to keep my mouth shut.” (#2, Male, 66)	“They just feel like, ‘I want to eat what I want to eat’…If you’re in a skilled nursing home, there are not too many things that you have control over. [Food] is one of the few things that they still have some control over.” (#8, Female, 50) “If you were to look at surveys of what is important to people in nursing homes, food is really going to always be very near or at the top. Restricted diets, I think, really contribute to a diminished quality of life in many cases.” (#6, Male, 47) “I think a lot of patients have a hard time with a true diabetic diet because it’s so unpalatable at times. My one patient with diabetes … was always like super happy with his incredibly bland meals but then was secretly ordering out so I think his actions spoke a little louder than his words.” (#4, Female, 32) “There’s a lot of [residents] with a lot of candy sitting in their drawers. But if that’s what they need for their quality of life I think that’s OK.” (#3, Male, 37) “I’ll call [one resident’s wife] and say, ‘He’s doing this and this,’ and she says, ‘Yeah, I understand.’ I’ll say, ‘You know this is what’s going to happen.’ And she says, ‘Yeah, I do but I’d rather have him happy.’” (#2, Female, 41)
Loss of independence due to diabetes	“[Diabetes] put me in a wheelchair and that’s been hard to get used to and it seems like I need more care to take care of myself than I used to. I used to be more able-bodied and be able to do more things for myself.” (#10, Male, 66) “Yeah I used to do a lot of activities, but now I slowed down and I don’t do as much activities as I used to do…I don’t have the energy for it… Participating in different sports like I want to, go out in crowds; I don’t do that no more.” (#3, Male, 74)	“Many of the people we get now are much sicker so… it's often hard to manage them because we want to give them freedom to eat what they want and to go out with their families or whatever while controlling their diabetes. It's difficult to manage both.” (#2, Female, 41)
Fingersticks and insulin injections: Strong Dislike	“The finger sticks…drive me nuts.” (#8, Male, 86) “Just painful, annoying…too much…they should find a way either before or after you eat and stick to monitor it. In between doesn’t make any sense to me.” (#2, Male, 66)	“There are a lot of folks who just really don't want finger sticks and you just really need to negotiate with them some sort of a schedule.” (#9, Female, 36) “The most frequent complaint that I get from them is the fact that they are pricked so often. Once they are on a stable regimen, we do try to cut down on the fingersticks.” (#10, Male, 44) “Some people are more vocal about it than others but I think if you ask most of my patients they would say that [they want] to be checked less frequently.” (#9, Female, 36)
Fingersticks and insulin injections: Annoyance/Don’t mind	“You never feel good about [fingersticks]…it’s an annoyance.” (#4, Male, 68) “One never gets used to them but they need to be done.” (#13, Male, 69) “It doesn’t hurt that much no, just a little dab and that’s it. I put up with it.” (#3, Male, 74)	“It depends…Some of them just don’t like needles. Some have been getting finger sticks for most of their lives so they’re used to it; it’s not a big deal. (#5, Female, 32)

We identified 3 sub-themes underlying diabetic VA NH residents’ lowered quality of life: dietary restrictions, loss of freedom due to diabetic symptoms, the burden of frequent fingerstick tests and insulin administration. (Table 2)

### Sub-theme 1a: Dietary restrictions

#### VA NH Residents

All NH residents disliked dietary restrictions and the diabetic diet, speaking at great length of foods they missed, “sneaking” food or “cheating” on their diet. Even the one NH resident who said that he did “not mind the diet” talked throughout the interview about food. One NH resident *(*#7, Male, 78) noted, *“The hardest thing is giving up the food that you used to enjoy; that is one of the hardest things about diabetes, giving that up.”*

Another common dietary theme among NH residents was “cheating” on their diabetic diet. One resident (#5, Male, 79) described how he would, *“eat other food and sometimes I order out, which I shouldn’t do because when I do I kind of hurt myself.”*

Other residents did not even attempt to follow the diabetic diet. After being asked whether there were specific foods that the participant wanted to eat, but could not, the participant *(#3, Male, 74)* responded, *“No I eat them anyhow, that don’t make no difference; I don’t worry about the diabetes.”* That same participant then went on to detail how he is, *“allowed one piece of cake, but sometimes I can have two or three pieces. Last Sunday, I had three pieces of cake and the woman came over and… she just told me, you have diabetes. I said ‘yes, but it’s all over on Sundays.’”* (Table 2)

#### VA NH Physicians

Although most of the NH physicians acknowledged that the VA NH residents did not like diabetes dietary restrictions, they appeared to underestimate how much NH residents disliked dietary restrictions. For example, one physician (#2, Female, 41) said, *“It’s often hard to manage them because we want to give them freedom to eat what they want and to go out with their families or whatever while controlling their diabetes and most of them are here because when they were out in the community they didn’t do a very good job of that on their own.”* (Table 2)

### Sub-Theme 1b: Loss of independence due to diabetes complications

#### VA NH Residents

Though not as intrusive as the diabetic diet, many NH residents cited loss of freedom due to diabetes complications as one of the biggest ways diabetes affected their quality of life. One 78 year old resident (#7, Male, 78) who had lost his foot as a result of diabetes said, *“Well, I lost my foot and I’m not able to walk around like I used to. Then it has affected my eyes; I can’t see like I used to see…You know it really makes a change in your life.”* A 70 year resident (#14, Male, 70) said, *“Two years ago they pulled my [boating] license because my eyes were going bad because of diabetes and that has been hard. It’s been hard. Every time I see a ship going down the bay I wish I was there.”* (Table 2)

#### VA NH Physicians

VA NH physicians were very conscious of their patients’ loss of independence and tried to minimize any resultant decreases in quality of life. For example, one physician (#6, Male, 47) said, *“I think the question is…how do you improve someone’s experience…and for the most part I think it’s trying to diminish the focus on the fact that they have a lot of medical issues and trying to focus on all other things around quality of life, so activities, food, contact with family and friends, those kinds of things.”* (Table 2)

### Sub-theme 1c: Fingersticks and insulin

#### VA NH Residents

NH residents had divergent opinions about fingerstick monitoring and insulin injections; some disliked the fingersticks, whereas others felt they were unobtrusive or accepted them as a part of having diabetes. One resident (#15, Male, 73) described himself as a, *“dog leashed to taking readings every day, seven days a week.”* At the other end of the spectrum, one resident (#7, Male, 78) said, *“I got so used to it; it’s a piece of cake now.”* (Table 2)

#### VA NH Physicians

VA NH physicians recognized the wide spectrum of opinions among their patients regarding fingersticks. One internal medicine doctor (#3, Male, 37) explained, *“Some people expect it and so some people are not bothered by it at all and some people absolutely hate it, and there’s people who are indifferent—so it’s very variable with people. I’d say most people don’t want it.”* (Table 2)

### Theme 2: NH Residents outlook and their level of engagement

#### VA NH Residents

The NH residents’ level of engagement in their own care appeared to correlate with their overall outlook, with about half of the residents maintaining a positive outlook on their situation and actively participating in their own care. For example, a 73 year old (#15, Male) who could no longer participate in his favorite sports as a result of his diabetes remained upbeat saying, *“You cope with it; you don’t get down or make yourself a cry baby; you get up and you fight it and you work it out. You do a little exercise downstairs with the PT people and try to walk…I don’t plan on tomorrow; it’s not here. There is no guarantee on tomorrow, only today. Live it to the fullest, period.”* (Table 3)

**Table 3:** NH Residents level of engagement and outlook on life

Themes	Representative CLC Resident Quotes	Representative Physician Quotes
Engaged and Positive	“The most important thing to me is getting myself well and… to realize how I am eating and is it a positive or negative cause and am I causing this.” (#12, Female, 78) “You’ve got to wake up. If you don’t wake up, hey, then they can only help you so far. So you have got to wake up. You have to take your medicine, you have to go to the different clinics and you co there because your body is not right and you’ve got to make it right.” (#12, Female, 78)	“It’s a team effort. I don’t dictate to my patients and I discuss with them and I think you–again, part of our job is to educate them, tell them what the complications are and, hopefully, they understand and will take it from there.” (#10, Male, 44) “Diabetes is something that [not] only the physician can treat, like most diseases you need the patient’s buy-in.” (#8, Female, 50)
Passive and Pessimistic	“I just take the medicine and hope for the best…and just do what they tell me to do.” (#10, Male, 66) “I don’t know [what] they say to me. They just write out prescriptions and give me medicine for what. That’s about it…There’s nothing I can do about [diabetes]; I can’t do nothing about it.” (#3, Male, 74)	“I think for overall, [my patient] didn’t care as much about what I did with his medicines, he was sort of more passive about things.” (#1, Female, 31)

Other NH residents had a more pessimistic outlook on life and were less engaged in their care. One (#14, Male, 70) said, *“As time has gone on it has made me a little more fatalistic about things; why not do it you are going to die anyway? And that is the truth; nothing you can say about it and nothing you can do about it. Your time is coming a lot quicker because you’ve got diabetes.”* (Table 3)

#### VA NH Physicians

The VA NH physicians noted this divide in patient engagement, though they did not attribute it to the patients’ outlook. For example, one geriatrician (#6, Male, 47) said, *“My experience with residents in the VA NH is that most are not terribly actively involved in the care of their diabetes. In other words, they leave it really to the providers and the nursing staff to manage it.”* (Table 3)

### Theme 3: How Being in a nursing home influences diabetes care

#### VA NH Residents

Many participants reported that living in a nursing home made managing their diabetes treatments easier. One resident (#7, Male, 78) explained, *“Here you have to [stick to your diet] because you’ve got all these dieticians and everything to watch me too and I have been able to keep it under control very well.”* (Table 4)

**Table 4:** How Being in a Nursing Home Influences Diabetes care

Factor	Representative CLC Resident Quotes
Diabetes care easier	“Well [the diet is] easy because I’m here; if I wasn’t here no, it would be hard.” (#4, Male, 68) “Just having somebody take care of your medications for you makes a hell of a difference because you would skip if you took as much medication as I did.” (#4, Male, 68)
Peer Influence: Negative	“I know there are a lot of different foods that other people get that I don’t get.” (#8, Male, 86)
Peer Influence: Positive	“You really want a lot of people there so you can get a good feedback; what are they eating and why are they eating this and can I get any other ideas from them and why are they not eating this…if it is helping them.” (#12, Female, 78)
Factor	Representative Physician Quotes
More information and more control	“The residents are a captive audience so to speak. We don’t have to worry about them not showing up to an appointment or having to remember, the system really takes over that role.” (#6, Male, 47) “I have a lot of collateral information and so just using that to help make decisions and kind of have an honest conversation with him not in a confrontational manner but just saying, ‘I know sometimes you might order food in; just take this into consideration.’” (#4, Female, 32)
Over treatment	“Versus outpatient, [nursing home residents] are going to get more fingersticks.” (#4, Female, 32) “The tough thing in a nursing home setting is that oftentimes it’s a structured environment so medications are given at a particular time and it’s hard when the patient can’t deliver their own insulin because if they’re not feeling hungry for breakfast they may still have been given an insulin shot before breakfast.” (#3, Male, 37)

Living in a NH also allowed residents to compare their treatments to those of other residents. These comparisons could be both positive and negative. One resident (#3, Male, 74) who reported feeling envious of other NH residents receiving different food said, *“Everybody else gets ice cream here more often than I do…It burns me up.”* Another resident (#10, Male, 66) said, *“You know you see the other people get it and you don’t- you can’t get it and you feel left out.”* (Table 4)

Other residents found having peers to be a positive influence, encouraging them to exemplify model behavior. As one resident (#7, Male, 78) described it, his *“goal is to be a good example for someone struggling with the same thing I have…If I show good effort or comply with the diet, it may encourage them.”* Another (#15, Male, 73) said, *“I talk to people and try to pass it on to them, what I have learned.”* (Table 4)

#### VA NH Physicians

Just as NH residents found managing their care easier in the VA NH, NH physicians also found managing their patients in nursing homes easier. Physicians reported that nursing homes gave them the advantage of having more control over and knowledge of their patients. For example, one physician (#5, Female, 32) said*, “In the [nursing home] you have a little bit more control over them. If you order the nurses to give them their medicines, order their sugars to be checked, it will get done with the exception of the patient who will occasionally refuse.”* (Table 4)

This ease of care, however, also had its negatives; some physicians stated that the regulated care in a nursing home often led to more monitoring and treatment than was optimal. As one physician (#1, Female, 31) explained, *“Your out-patients, they might be checking their finger sticks every once in a while; really trying to reproduce that but then there’s always that temptation to check more because you are in a setting like a hospital that could facilitate that pretty easily.”* (Table 4)

## Discussion

Our study found that VA NH residents and their physicians viewed diabetes and diabetes treatments as burdensome, lowering residents’ overall quality of life. Based on the NH residents’ and physicians’ interviews, the factors we identified as negatively affecting residents’ lives included the diabetic diet, loss of freedom due to diabetic symptoms and the burden of frequent fingerstick tests. We also found that most physicians experienced a constant tension between wanting to minimize burdens by liberalizing diets and decreasing fingerstick monitoring versus providing more intensive glycemic treatments to decrease the risk of vascular complications.

One of our more surprising findings was how burdensome dietary restrictions were for NH residents. Without exception, the residents talked at length about food—the food they did not like, the food they used to eat and the food they should not eat but still do. Even the one participant who stated that he/she did not want to talk about the diabetic diet, subsequently brought up the topic of food several times. Our results suggest that although guidelines suggest minimizing dietary restrictions in older adults with diabetes in nursing homes [13–16], these restrictions may be common and substantially detract from NH residents’ quality of life.

Although there was good concordance between NH residents and their physicians regarding most aspects of treatment, physicians appeared to substantially underestimate their patients’ feelings about diet. Many physicians, citing the difficulties of a restrictive diet, stated that they tried to give their patients a more lenient diet. However, patients of these same physicians mentioned “sneaking” food or “cheating” on their diets. Thus, while NH residents uniformly wanted a more permissive diet, physicians often felt that some dietary restrictions are needed. Some physicians noted that most residents came to the nursing because they had managed their diabetes poorly in the community, suggesting that although NH residents don’t want dietary restrictions, they may be better off with dietary restrictions. However, since Cochrane systematic review showed no evidence that specific diets improve diabetes outcomes and dietary restrictions appear to impose substantial burdens on NH residents, more permissive diets may be most appropriate in most NH residents with diabetes [17].

A second notable finding was the relationship between NH residents’ attitude and engagement in their own care [18–21]. The NH residents reporting a more positive outlook also reported being more engaged and were the same residents trying to set a good example for their fellow NH residents. The NH residents with a more negative outlook took a more “hands off” approach and tended to be the same residents who reported being jealous of what their peers got to eat.

One interpretation of our findings is how NH residents and physicians view the structured NH setting differently. For many NH residents, although the structure and resources of the NH setting made diabetes care easier, the NH setting decreased autonomy, leading to more restrictive diets and more interventions that they did not like (e.g. fingerstick monitoring). For NH physicians, the NH setting allowed for more control and information about their patients. This interpretation reinforces the importance of communication between NH residents and physicians so that evolving resident preferences are continually incorporated into NH care plans. Further, since NHs are a setting where even intrusive care plans can be easily implemented, NH physicians should continually strive for minimally intrusive care plans that are concordant with NH resident preferences.

To our knowledge, no previous qualitative study has focused on diabetic NH residents’ and their physicians’ perspectives on the burdens of diabetes and diabetes treatments. One systematic review noted “a severe lack of research” resulting in a “lack of the voice of the nursing home resident [22].” Although previous qualitative studies have focused on nursing home residents’ level of knowledge about diabetes,[7] our study is the first that focuses on nursing home residents perceptions of the burdens of diabetes and diabetes treatments.

Previous studies focusing on community-dwelling older adults have found that although diabetes treatments and monitoring were burdensome, patients had a strong desire to maintain independence which generally led them to adhere to physicians’ recommendations [5, 6, 21, 23]. Compared to these previous studies, our study suggests that the burdens of dietary restrictions are even more onerous for older adults in NHs than older adults living in the community. Further, while community-dwelling older adults focus on maintaining independence as a primary reason for being adherent to care recommendations, NH residents who have already lost their ability to live independently focus on other reasons such as being a good example to remain engaged in their care [24, 25]. However, other NH residents become disengaged and fatalistic, “cheating” on their diets and allowing the NH staff to dictate nearly all aspects of their care.

Our results must be interpreted in the context of the limitations of our study. Our study was conducted at one VA NH; thus, our results may not be representative of non-VA NHs or NHs in other parts of the country. Further, our sample was predominantly male; it is unclear how much of our results would hold for older women in nursing homes. Since the interviews took place at the VA CLC, our participants may have felt influenced to speak more positively about their treatment experiences, despite being told that the interviews were confidential and would have no impact on their treatment. We also interviewed only those who were willing and able, potentially leaving out participants who were sicker and less satisfied with their care. Despite these limitations, we believe this study adds depth to our understanding of the burdens and benefits of diabetes treatments experienced by older NH residents.

## Conclusion

In conclusion, we found that VA NH residents with diabetes were substantially burdened by their diabetes and diabetes treatments. NH residents focused on dietary restrictions and fingerstick monitoring as the most burdensome interventions. The physicians for these patients recognized dietary restrictions and fingerstick monitoring as burdensome; however, physicians appeared to underestimate how burdensome dietary restrictions are for NH residents. Since systematic reviews have found little evidence that dietary restrictions improve outcomes, fewer dietary restrictions may be appropriate and lead to lower treatment burdens for NH residents with diabetes.

## Additional files

Additional file 1:
**Resident Interview Guide.** (DOC 40 kb) Additional file 2:
**Provider Interview Guide.** (DOC 35 kb)

## Abbreviations

CLC: community living center
DM: diabetes mellitus
HbA1c: hemoglobin A1c
NH: nursing home
VA: veterans affairs
