# Supplementary material for: “Everyone else gets ice cream here more often than I do—It burns me up” - Perspectives on Diabetes Care from Nursing Home Residents and their Doctors
Source: BMC Geriatr. 2016 Jan 26;16:28. doi: 10.1186/s12877-016-0199-0 (PMC4729138; doi:10.1186/s12877-016-0199-0)
Supplement: Additional file 1: — Resident Interview Guide. (DOC 40 kb) [file 12877_2016_199_MOESM1_ESM.doc]

CLC Resident Interview Guide

Glycemic Control and Geriatrics Outcomes in National Sample of VA CLC Residents

Semi-Structured Interview Guide

| Interviewer: | Interview date: |
| --- | --- |
| Interviewee ID: |

Introductory Script

**Thank you for agreeing to speak with me today. We are interested in learning more about the things that are important to you when it comes to the care you are getting for your diabetes. We hope that what you tell us will help us improve the way doctors, nurses and pharmacists care for older patients with diabetes.**

1. Patient’s General Perception of Own Health

**I would like to start by asking you some questions about your health.**

1. How are you feeling right now?
2. Tell me about your life with diabetes.
3. Did you get diabetes before coming to the nursing home or were you diagnosed with diabetes after entering the nursing home?
   - (If diabetes before NH) What were you doing to manage your own diabetes before coming into the nursing home? [Probe to get a sense of how active and engaged they were in managing their diabetes prior to the NH]
   - (If diabetes before NH) How is your diabetes being managed now?
   - (If diabetes before NH) Has your experience managing your own diabetes before the NH affected how you want your diabetes treated now that you’re in the NH? How?
4. General Patient perceptions of Diabetes Treatment
5. What do you think are the worst things that could happen to you because of your diabetes?
6. What are your reasons for getting diabetes treatment?
7. Tell me about who has influenced you to get diabetes treatment.
   - Probe 1: Ask about the role of family, doctors, nurses, other health professionals, other residents)
   - Probe 2: Has the influence been a positive or negative experience?
8. Do you feel that you have control of your diabetes treatments? Who else has control over your diabetes treatments? (Probe doctors, nurses, other health professionals, family, other residents)
9. What are the things that are important to you about your diabetes care?

**I would like to ask you about the treatments you currently receive for your diabetes.**

1. Specific Treatments and Perceived Burdens of Treatments
2. Do you get finger sticks? (If so, how often do you get them? How do you feel about the finger sticks?)
3. Do you take medicines by mouth for your diabetes? (If so, what is your daily routine with these medicines? Do you feel like you’re taking not enough medicines, too many medicines, or the right amount of medicines?)
4. Do you take insulin? (If so, what is your daily routine with insulin?)
5. Are you on a special diet because of your diabetes? (If so, could you tell me what your diet is like?
   - Probe 1: Does the CLC provide the foods you need to eat to follow your diabetes diet?
   - Probe 2: Are there specific foods you want to eat but don’t or can’t because of your diabetes?
6. Are there other treatments you’re getting for diabetes?
7. What do you think about the treatments you are receiving for your diabetes? If patients don’t address whether the treatments are helpful or not, then ask, “Do you think the finger sticks/medications/special diets are helping you?”
   - Has diabetes treatments changed your life? In what way?
8. What do you think would make your diabetes care better?
9. Diabetes Care Goals
10. How important is it for you to have your diabetes treated? What is the reason you feel the way you do?
11. What do you feel is the most important benefit from treating your diabetes?
12. What are the hardest things about having diabetes?
13. What are the hardest things about diabetes treatments?
    - Probe: (If respondent doesn’t bring up burdens of treatment: Since some treatments are hard and bothersome, do you think your diabetes treatments are worth it?)
14. Additional Information

**What haven’t I asked you that you think is important for me to know about your diabetes and the care you are receiving?**
