# Supplementary material for: “Everyone else gets ice cream here more often than I do—It burns me up” - Perspectives on Diabetes Care from Nursing Home Residents and their Doctors
Source: BMC Geriatr. 2016 Jan 26;16:28. doi: 10.1186/s12877-016-0199-0 (PMC4729138; doi:10.1186/s12877-016-0199-0)
Supplement: Additional file 2: — Provider Interview Guide. (DOC 35 kb) [file 12877_2016_199_MOESM2_ESM.doc]

CLC Provider Interview Guide

Glycemic Control and Geriatrics Outcomes in National Sample of VA CLC Residents

Semi-Structured Interview Guide

| Interviewer: | Interview Date: |
| --- | --- |
| Interviewee ID: | Type of Clinician: |

Introductory Script

**Thank you for agreeing to speak with me today. We want to help make glycemic control decisions more patient-centered for older veterans with diabetes. We want to find out what factors you consider when you recommend specific treatments and glycemic targets for individual patients. We hope that what you tell us will help us improve how diabetes care is provided for CLC residents.**

1. DM Treatment Benefits and Burdens

**I would like to ask you about how you make glycemic treatment decisions for your older patients.**

1. How do you feel about taking care of patients with diabetes? If required, probe: Are they some of your more challenging patients? Are they some of your more rewarding patients?
2. How do you approach a patient who gets diagnosed with diabetes while residing in the nursing home? How different is this from how you would work with a patient who enters the nursing home with a diagnosis of diabetes?
3. How do you try to engage residents with diabetes in glycemic treatment decisions?
4. What are the things you consider when making glycemic treatment decisions?
5. Do you ever feel like you should treat sugars more aggressively? Does this feeling come from patients? Families? Nurses? Other staff? Guidelines? Anyone else?
6. Do you ever feel like you should treat sugars less aggressively? Who encourages you to treat sugars less aggressively? Patients? Families? Nurses? Other staff? Guidelines? Anyone else?
7. Who do you feel makes the decisions about glycemic control? You? The patient? The patient’s family? Someone or something else?
8. What do you feel is the primary goal of glycemic control in CLC residents with diabetes? How come?
9. What aspect(s) of diabetes and diabetes treatments do you think is hardest for your patients?
10. What kind of special consideration(s), if any, do you take into account when caring for an *older* patients with diabetes?
11. Specific Treatments

**I would like to ask you now about the burdens of some of the diabetes monitoring and treatment.**

1. Do you feel that most patients are bothered by finger stick monitoring? How come?
2. Do you feel that most patients are bothered by insulin injections? How come?
3. Do you feel that most patients are bothered when restricted to a “diabetic diet.” How come?
4. How to Improve Reaching Health Care Goals for Patients

**I would like to ask you about how you think you could help your patients better reach their health care goals.**

1. What do you think would help your patients better manage his/her diabetes? Are there things that nurses, aides, families and doctors could do to help older patients with diabetes manage their sugars better?
2. What things keep patients, nurses, aides, families and doctors from improving the glycemic treatment care of older patients with diabetes?
3. Have you cared for older patients with diabetes living in the community? If so, do you feel it is easier or harder to care for a patient with diabetes in the nursing home versus living independently in the community? Why?
4. Additional Information

**What haven’t I asked you that you think is important for me to know?**
